# Breaking the reproductive barrier of divergent species to explore the genomic landscape

**DOI:** 10.3389/fgene.2022.963341

**Published:** 2022-09-23

**Authors:** A. Gilles, Y. Thevenin, F. Dione, J.-F. Martin, B. Barascud, R. Chappaz, N. Pech

**Affiliations:** ^1^ Aix Marseille University, INRAE, UMR 1467 RECOVER, Centre Saint-Charles, Marseille, France; ^2^ CBGP, Montpellier SupAgro, INRA, CIRAD, IRD, Université Montpellier, Montpellier, France

**Keywords:** speciation, SNP, RAD sequencing, hybridization, genomic introgression, transcriptome

## Abstract

**Background:** Climate change will have significant consequences for species. Species range shifts induce the emergence of new hybrid zones or the spatial displacement of pre-existing ones. These hybrid zones may become more porous as alleles are passed from one species to another. Currently, hybridization between highly divergent species living in sympatry seems extremely limited. Indeed, this phenomenon involves breaking two barriers. The first is the pre-mating barrier, related to the reproductive phenology of the two species. The second is the post-zygotic barrier, related to the genetic divergence between these species. Here, we were interested in identifying new hybridization patterns and potential implications, especially in the context of environmental modifications.

**Methods:** We sampled *Telestes souffia* and *Parachondrostoma toxostoma* wild specimens from different locations across France and genotyped them for SNP markers. We identified discriminant loci using F1-hybrid specimens and parental species and performed principal component analysis and Bayesian model-based clustering to analyze phylogenetic information. Furthermore, we assessed deviation in allele frequency from F1 to F2 and for Hardy–Weinberg equilibrium for F2 and assessed gene function associated with two F2 cohorts.

**Results:** We demonstrate that by breaking the ecological barrier, massive introgressive hybridization is possible between two endemic lineages of Cyprinidae belonging to two distinct genera. For both cohorts studied (=2 cm and >2 cm), a large majority of loci (>88%) presented no deviation in allele frequency and no departure from the Hardy–Weinberg equilibrium. For individuals beyond the 2 cm stage, two phenomena were observed. The first was an allelic imbalance in favor of *P. toxostoma*, for some genomic regions, with genes involved in developmental regulatory processes, cytoskeletal organization, and chromosome organization. The second was an excess of heterozygous loci coupled with an equilibrium of allelic frequencies for genes involved in immune response and kidney/liver development. Moreover, the 2 cm-sized specimens with high mortality yielded a particular genomic signature.

**Conclusion:** Our study displayed important results for understanding the early stages of hybridization between divergent lineages and predicting the emergence of future hybrid zones in the wild. Moreover, this hybridization generates a wide spectrum of hybrids that are a potential source of important evolutionary novelties.

## 1 Introduction

As early as 1948, Anderson (1948) hypothesized that the impact of anthropogenic changes would affect habitats in such a way that hybridization events would be more frequent and hybrid survival would be greater. Although some authors (Lewontin and Birch, 1966) have described hybridization as a source of variation allowing adaptation to new environments, a predominantly negative view of hybridization has long prevailed, illustrated by terms such as ‘extinction by hybridization,’ ‘genetic pollution,’ or ‘evolutionary dead end’ (Rhymer and Simberloff, 1996). However, hybridization can generate evolutionary novelties, such as transgressive segregation at a much faster rate than intra-specific adaptation, to transfer new alleles (adaptive introgression of alleles), allowing, for example, the evolutionary rescue of a lineage living in extreme pollution conditions (Oziolor et al., 2019), or new species induction (speciation by hybridization) in new environments, either by allopolyploidy (when the ploidy level of the hybrid populations is equal to the sum of the chromosomes of each parent species) or by homopolyploidy (when the ploidy level of the hybrid populations and that of the parent species do not change). This paradigm shift in hybridization is due to increased research in the field. Indeed, our knowledge of hybridization in the wild has increased considerably and has led to a better understanding of the mechanisms involved and its evolutionary potential (Runemark et al., 2019).

However, the ability of two species to hybridize and the fate of hybrid individuals is difficult to predict. Indeed, it is unclear how to predict future hybridization behaviors for species that do not currently hybridize. Furthermore, there may be genomic porosity between phylogenetically distant species that are hidden by current environmental conditions. Owens and Samuk (2020) modeled the effects of climate change on hybrid zones and showed that hybrid zones become increasingly porous as climate change-adapted alleles are passed from one species to another, and that this effect is stronger when climate change is greater. Other authors have proposed models to predict the incompatibility of hybrids with the parental species that generated them (Schumer et al., 2015).

There is growing recognition that climate change will reshape species assemblages and shift spatial, temporal, and behavioral reproductive barriers (Chunco, 2014). Climate change leads to shifts in species ranges that can result in the displacement of pre-existing hybrid areas, such as for the two chickadee species *Poecile atricapillus* and *Poecile carolinensis* (Taylor et al., 2014), or new hybrid areas for the two hare species *Lepus timidus* and *Lepus europaeus* (Jansson et al., 2007). Thus, climate change may alter not only the respective densities of species present in the environment but also the sex ratio within each species. In both cases, heterospecific crosses can occur and lead to the existence of hybrids. Under these conditions, some hybrid combinations could emerge in these new environments and be selected positively or negatively (exogenous selection). However, for this to happen, it is first necessary for the genomic compatibility between the species to be possible (endogenous selection) and, second, to overcome the barrier of F1 individuals either by backcrossed specimens or by F2-hybrids.

Many studies (Edmands, 2002) have investigated whether there is a correlation between the genetic divergence of parental species and their level of reproductive incompatibility (pre- or post-zygotic). In this case, the greater the phylogenetic divergence, the lower the genetic compatibility, and vice versa. Some authors (Orr and Turelli, 2001) have proposed the ‘snowball’ model to model the evolution of genetic incompatibility with genetic divergence or time and showed that the expected number of hybrid incompatibilities rose exactly with the square of time. This was empirically verified in two scientific papers, one on the genus *Drosophila* (Matute et al., 2010) and the other on the genus *Solanum* (Moyle and Nakazato, 2010). Moreover, this genetic incompatibility, which increases with genetic divergence, is accompanied by an increase in transgressive phenotypes due to divergent alleles from the two parental species (Stelkens and Seehausen, 2009; You and Xu, 2021), leading to non-additive effects by over-dominance, in addition to epistatic interactions, such as the modification of the expression of a gene by other genes (Stelkens and Seehausen, 2009). In the case of Cichlidae of the Haplochromine group, genetic divergence between species explains 52% of the variation in transgressive traits for F1-hybrids and 78% for F2-hybrids (Stelkens and Seehausen, 2009). Thus, hybridization between two different genera is rare, but such cases exist in some Teleost families, such as Cyprinidae (Scribner et al., 2000). The majority of these hybrids are the result of crossing between an endemic species and a closely related introduced genus, as in the case of *Chondrostoma nasus* and *Parachondrostoma toxostoma* resulting in fertile hybrids (Costedoat et al., 2007), or between two introduced species, as in the case of *Rutilus rutilus* and *Abramis brama* (Hayden et al., 2014; Konopiński and Amirowicz, 2018) yielding mostly F1-hybrids. Finally, some rare cases involve a natural hybridization of two endemic species, as in *Achondrostoma oligolepis* and *Pseudochondrostoma duriense* (Pereira et al., 2014). As Canestrelli (Canestrelli et al., 2016) aptly pointed out: ‘Not surprisingly, species of ancient divergence and with a long-lasting history of sympatry have had ample opportunity to evolve strong pre-mating reproductive barriers’.

Given the existence of hybridization phenomena, we were interested in identifying new ones, especially in the context of environmental modifications. Moreover, hybridization is a common phenomenon in fishes in general and cyprinids in particular (Smith 1992; Dowling and DeMarais, 1993). We, therefore, proposed the following question: To what extent is it possible to qualify and quantify the potential porosity of evolutionary barriers between two species that may be very divergent? In order to answer this question, we were interested in two endemic species of southern France, *Parachondrostoma toxostoma* (Vallot, 1837) or toxostome and *Telestes souffia* (Risso, 1826) or blageon. These two species live in sympatry and are phylogenetically distant with a time of divergence estimated at 15 million years, based on the molecular marker whole cytochrome b (Doadrio and Carmona, 2004; Perea et al., 2010). These two species have an extremely low natural hybridization rate since only two hybrid specimens have been detected in the wild (Dubut et al., 2010; Dubut et al., 2012), and such hybrids had been identified in the past, which led to the invalidation of the species *Chondrostoma rysela* (Günther 1868 in (Roule, 1925; Kottelat, 1997)). The rarity of these hybrids can be explained not only by very strong genetic incompatibility due to phylogenetic distance but also by the existence of ecological barriers. Indeed, *P. toxostoma* specimens reproduce (single spawn) essentially from April until the beginning of May in the range of water temperature from 11 to 13°C in the Buech tributary of Durance, France (Costedoat et al., 2007), whereas *T. souffia* specimens reproduce (single spawn) during the month of June, in water temperatures from 10 to 14°C. Warmer temperatures and low water levels in May–June will cause the shift of breeding season to the earlier end of March and beginning of May. The most impacted species will be the *T. souffia* because June would not present the appropriate conditions for reproduction. Moreover, the densities of the two species are reversed along an upstream–downstream thermal gradient (mostly *T. souffia* upstream, *P. toxostoma* downstream). For this study, we investigated 369 specimens represented by field-sampled (*T. souffia* and *P. toxostoma* ‘pure’ specimens) and laboratory-bred individuals (*P. toxostoma* ‘pure’ specimens, F1-hybrids, F2-hybrids, and backcrossed specimens), and 5,289 single nucleotide polymorphisms (SNPs) based on the *P. toxostoma* transcriptome.

## 2 Materials and methods

### 2.1 Presentation of the species and populations used

The *Telestes souffia* or blageon is a species of about 15 cm with a purplish stripe on the flanks, especially in males. It is protected but considered to be of least concern on the IUCN global red list (2008 assessment, https://www.iucnredlist.org/species/61397/12461824) and the red list of freshwater fishes of metropolitan France (2019). We used specimens belonging to the lineage *T. s. souffia,* which is specific to the Rhône and Mediterranean coastal river basins (from the Hérault River to the Var River) (Dubut et al., 2012). The *Parachondrostoma toxostoma* or toxostoma is a species with a tapered body, 15–25 cm long, olive-green in color, with light-colored sides showing silver reflections and a dark band that stands out, particularly during the breeding season. It is protected and is considered vulnerable on the IUCN world red list (2006 assessment, https://www.iucnredlist.org/species/4795/11095903) and near threatened on the red list of freshwater fishes of metropolitan France (2019). Its range extends from the southwest (the Adour–Garonne basin) to the southeast (the Rhône and Mediterranean coastal river basins) of France. Both species are diploid and have an identical number of homologous chromosomes (2n = 50).

### 2.2 Wild samples and specimen crosses

**FIGURE 1:**
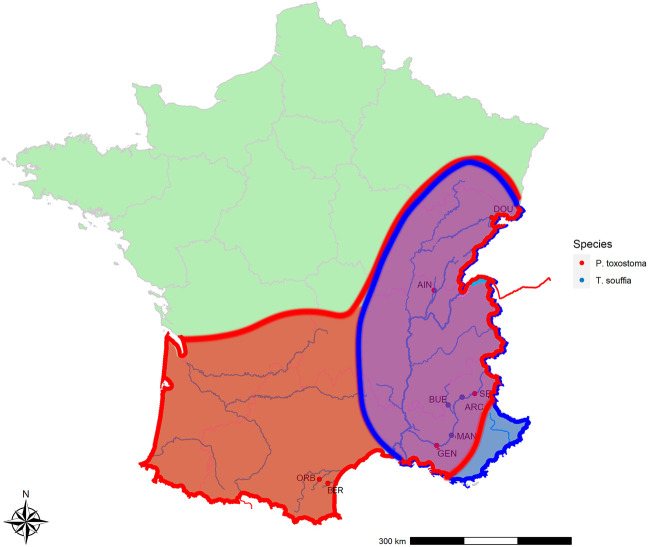
Species distribution and collection sites. In red *P. toxostoma* sites, in blue T. souffia sites.

To calibrate SNP discriminant loci, we used wild specimens sampled in different locations (see Figure 1 and Table 1). For the *T. souffia* species, we selected four populations from the Rhône basin, especially in the Durance River: ARC_Ts (Archidiacre station on Durance River Lat. 44.475,719 Lon. 6.111,014), MAN_Ts (Manosque on Durance River Lat. 43.8,402,500 Lon. 5.8,506,111), BUE_Ts (Buech River Lat. 44.344,493 Lon. 5.770,268), and AIN_Ts (Ain River Lat. 46.264,383 Lon. 5.429,392), which were previously described (Dubut et al., 2012). For *P. toxostoma*, because of natural hybridization with *Chondrostoma nasus*, we selected three populations in parapatry from the Rhône basin, with no sign of hybridization—DOU_Pt (Doubs River Lat. 47.4,812,500 Lon. 6.8,360,556), SEP_Pt (Serre-Ponçon dam Lat. 44.536,424 Lon. 6.417,518), and AIN_Pt (Ain River Lat. 46.264,383 Lon. 5.429,392)—and two in allopatry—BER_Pt (Berre River Lat. 43.032721 Lon. 2.831,686) and ORB_Pt (Orbieu River Lat. 43.101,508 Lon. 2.623,265)—which were previously described (Costedoat et al., 2007; Corse et al., 2015).

**TABLE 1:** SNP characteristics mapping.

Level of SNP detection on *P. toxostoma* transcriptome	Loci number	Number of conserved loci	Number of SNPs	Groups	Tissue collection year	Number of individuals	Number of unique individuals	Number of sequencing replicates	Number of extraction replicates	Sequencing run	Number of reads
70% (within populations and between populations)	89,787	10,099 (11.25%)	56,283 (5,289 variable sites)	ARC_Ts	1996	3	3			run1	7,717,035
				MAN_Ts	1996	4	4			run1	8,073,878
				ORB_Pt	2007	5	5			run1	12,728,611
				AIN_Pt	2000	5	5			run1	11,393,464
				F1_Hy_R1	2015	19	14	5		run1	181,875,041
				F2_Hy	2021	213	204	4	5	run2	1,597,097,231
				BK_Hy	2015	15	10	1	4	run1	197,879,261
				AIN_Ts	1996	12	11	1		run1	46,468,500
				DOU_Pt	1996	5	5			run1	33,934,941
				SEP_Pt	1997	7	6	1		run1	24,326,823
				LAB_Pt	2021	33	33			run2	674,783,611
				F1_Hy_R2	2021	9			9	run2	42,338,828
				GEN_Pt	2011	5	5			run2	195,101,630
				BUE_Ts	2021	24	24			run2	176,614,316
				BER_Pt	2007	10	10			run2	40,999,443

*P. toxostoma* populations: AIN_Pt for Ain River, DOU_Pt for Doubs River, ORB_Pt for Orbieu River, BER_Pt for Berre River, GEN_Pt for genitors, LAB_Pt for laboratory specimens, SEP_Pt for Serre-Ponçon dam. *T. souffia* populations: AIN_Ts for Ain River, ARC_Ts for Archidiacre River, BUE_Ts for Buech River, MAN_Ts for Manosque on Durance River. F1_Hy_R1, first run of F1 laboratory specimens; F1_Hy_R2, second run of F1 laboratory specimens; F2_Hy. F2 specimens; BK_Hy, backcrossed laboratory specimens between F1 and *P. toxostoma* laboratory specimens.

In 2011, 14 F1-hybrids specimens were obtained by *in vitro* fertilization of 50 eggs and semen between one female T. souffia (from the population BUE_Ts) and one male P. toxostoma (from the population GEN_Pt, inhabiting the Durance River). Free-swimming larvae were first housed in small aquaria (40 × 25 × 15 cm); after 4 months, the fish were transferred into larger aquaria for growth (100 × 60 × 40 cm). Fish were fed live *Artemia* nauplii, complemented with commercial fish food. The P. toxostoma laboratory stock (LAB_Pt) was obtained by crossing one female (population GEN_Pt from the Durance River at Pertuis) and two males (population GEN_Pt from the Durance River at Pertuis). In 2014, nine backcrosses were obtained by crossing one female P. toxostoma (3 years old) from the laboratory stock and three male F1-hybrids (3 years old). Furthermore, 10 F2-hybrids were obtained in 2014 and 194 in 2018 by crossing eight F1-hybrid specimens (3 and 7 years old) by crossing two females and six males (random mating from the F1 population).

### 2.3 Molecular protocol

In total, 369 individuals (including 12 DNA extraction replicates and 18 tissue replicates on specimens alive 4 years later, that constituted 8.13% of replicates) belonging to the two species and their hybrids (F1-hybrids, F2-hybrids, and backcross) were genotyped for SNP markers by first digesting genomic DNA with PstI restriction endonuclease as previously described (Brazier et al., 2021) (Supplementary Material) with the modifications described in Supplementary Figure S1.

We sequenced the mitochondrial DNA (1,140 bp of the cytochrome b) for the whole hybrid specimens, corresponding to only two haplotypes: *T. souffia* for F1-hybrids and F2-hybrids and *P. toxostoma* for backcross (Supplementary Data S1).

A sequencing Illumina Hiseq 2000 1 × 100 bp (Single End) run was generated with 5% Phi-X Spike-in by Cornell University (https://www.biotech.cornell.edu/facilities-brc). Then, two sequencing Illumina NovaSeq 2 × 150 bp (Pair End) runs were generated with 10% Phi-X Spike-in by NOVOGENE (https://en.novogene.com). The quality of the three sequencing runs was checked independently using FastQC, and the demultiplexing step was performed using the ‘process_radtags’ program implemented in the Stacks software (2.53) (Catchen et al., 2013; Rochette and Catchen, 2017). All the samples were analyzed in single-end and submitted to SRA (Sequence Read Archive, https://www.ncbi.nlm.nih.gov/sra, BioProject: PRJNA863842Submission ID: 30065804-30066172). The demultiplexing step was performed as follows: reads with an uncalled base were removed, a quality Phred score = 33 was used, and reads were truncated to 80 bp (Min-Max). The corresponding command was:

#### 2.3.1 Process_radtag-f/raw_fastq-o/sample/-b/pst_barcodes.csv–inline_null–renz_1 pstI -r -c -q -t 80 –len_limit 80

We conserved 99% of reads per individual. We used the genome mapping option of Stacks software (2.53) on the *P. toxostoma* transcriptome (Ungaro et al., 2017). The transcriptome displayed orthologous ENSDARG annotation (Ensembl, https://www.ensembl.org, *Danio rerio*, gene) because it corresponded to a model system belonging to the same family, Cyprinidae. We used the genome nomenclature (ENSDARG) rather than transcriptome nomenclature (ENSDART) because we used the chromosome and gene position of *D. rerio* for mapping the discriminant SNPs between the two species *P. toxostoma* and *T. souffia*. Then, we used the ‘ref_map.pl’ program in the Stacks software (2.53). The corresponding command was:

#### 2.3.2 Ref_map.pl -T28–popmap/popmap.csv -o/stacks/--samples/sample/

The program consisted of four principal steps (Paris et al., 2017): 1) Short read alignments were performed using BWA-MEM (Li and Durbin, 2009; Li, 2013) on the *P. toxostoma* transcriptomes. 2) The output data in SAM file format was sorted and converted to BAM file format using the software SAMtools (Li et al., 2009). 3) Then, SNP scoring and genotype identification was performed on reads alignment for each sample using the ‘Gstacks’ program of the Stacks software. 4) Finally, we conserved only the loci shared between 70% of the individuals within and between groups using the Populations software (Catchen et al., 2013; Rochette and Catchen, 2017).

### 2.4 Analysis of single-nucleotide polymorphism data

Selecting useful SNPs from Restriction-site Associated DNA-sequencing (RAD-sequencing) constitutes an important step in data analysis (Díaz-Arce and Rodríguez-Ezpeleta, 2019). Thus, we developed an approach to identify and select discriminant loci using F1-hybrid specimens and parental species (using different populations). The initial dataset consisted of 369 individuals and 5,289 SNPs. The individuals corresponded to five groups: wild populations of *P. toxostoma* (70 individuals from seven populations), wild populations of *T. souffia* (43 individuals from four populations), and farmed individuals—F1 (28 individuals), F2 (213 individuals), and backcrossed (15 individuals).

We defined the Euclidean distance between two specimens or replicates as the square root of the sum of squares of genotypic values (0 for homozygous *P. toxostoma*, 1 for heterozygotes, and 2 for homozygous *T. souffia*) based on the whole loci. The distribution of the 67,896 pairwise distances showed a multimodal distribution with three modes. We hypothesized that the first mode, corresponding to the 34 lower distances, included only the DNA and/or extraction replicates. Considering this criterion, we removed 18 specimens: twelve corresponded to DNA replicates (100%), four corresponded to extraction replicates (33%), and two were not replicates and could correspond to contaminated samples (i.e., genetically similar individuals). However, for the 18 extraction replicates, twelve (66.66%) did not belong to the first distribution but were closer to their replicate than any other individual: 9/9 F1 (100% retrieved), 3/4 F2 (75%), and 0/5 backcrossed (because the five specimens clustered together).

Polarization of the alleles was performed. To do this, the reference allele (coded as 0) was chosen as the nucleotide with the highest proportion of *P. toxostoma* in wild populations. A particular case involves an SNP present only in a single homozygous state in *P. toxostoma* populations (coded as 0) and present in a single homozygous state (different) in *T. souffia* populations (coded as 2). In the following, such SNPs are referred to as diagnostic SNPs.

The definition of a diagnostic SNP was relaxed for discriminant SNPs by defining a score associated with each SNP as the mean average of the proportion of homozygous genotypes (0/0) in *P. toxostoma* individuals and the proportion of homozygous genotypes (1/1) in *T. souffia* individuals. This score varied between 0 and 1 and was exactly equal to 1 in the case of a diagnostic SNP. In the following, an SNP will be considered discriminant if its score is strictly higher than a fixed threshold (0.9). This threshold was chosen from the empirical score distribution (see Section 3).

From a theoretical point of view, an F1 genotype for a diagnostic SNP that presents a sum of alleles equal to 1 is heterozygous (0/1). To verify this on our empirical data, a volcanic eruption plot was generated considering discriminant SNPs. In this plot, a point refers to an SNP. The abscissa (x) shows the mean genotype score calculated on the F1-hybrid specimens, and the ordinate (y) shows the standard deviation of the genotype score calculated on the F1-hybrid specimens. The point of coordinates (x = 1, y = 0) indicates perfect agreement with the theory. A deviation from the value x = 1 was tested using the classical Student’s t-test (type I error set at 0.05). All SNPs which deviated significantly from 1 were removed. When several SNPs had the same coordinate, the size of the point was proportional to the number of SNPs.

### 2.5 Principal component analysis

PCA was performed on the data corresponding to the selected 641 SNPs and 350 individuals. The original data corresponded to (Cij), 1 ≤ *i* ≤ *n*, 1 ≤ *j* ≤ *p* where Cij was equal to 0, 1, or 2 and expressed the genotype of the *j*th SNP for an individual i. Here, *p* refers to the number of SNPs selected using previous procedures. When a value Cij was missing, it was imputed by the mean genotypic value calculated on individuals belonging to the same group as the individual i. The normalization proposed by Patterson et al. (2006) was used before performing PCA. Moreover, the test of significance for axes was performed following the procedure of Patterson et al. (2006).

### 2.6 Bayesian procedure

We used a Bayesian model-based clustering algorithm implemented in the software STRUCTURE version 2.3.4 (Hubisz, Falush, Stephens, & Pritchard, 2009). Individuals in the sample were assigned to K populations or a mix of populations if their genotypes indicated that they were admixed (i.e., for F1, F2, and backcross). For the ancestry model, we used the option ‘admixture model’ because different alleles for hybrid specimens (F1, F2, and backcross) could be inherited from different species populations. Furthermore, we selected the option ‘allele frequencies correlated’. The burn-in length was set to 100,000 followed by 1,000,000 iterations using a Markov Chain Monte Carlo as recommended previously (Pritchard et al., 2000). These analyses were carried out with K = 2 (i.e., two species).

### 2.7 Genomic combination of F2-hybrid specimens

Considering the 204 F2-hybrid specimens, we observed important mortality (38/204, 18.63%) occurring during 2 weeks, corresponding to a 2-cm size. Because this event could correspond to a specific post-zygotic barrier underlined by a particular genomic combination, we first analyzed the cohort of 166 F2 individuals who survived (>2 cm), and second, the cohort of 38 F2 individuals who died very young (=2 cm). For both cohorts, we tested two hypotheses related to the gametic and zygotic phases, respectively, during the formation of the F2 genome. Our study corresponded to a segregation distortion analysis (Fu et al., 2020).

The first hypothesis was related to allelic transmission from F1 to F2; if allele transmission is independent of allele origin, then H0_1 (hypothesis H0 number 1): the mean genotype value is equal to 1, for F2-hybrid specimens as for F1s. Otherwise, the genotype mean value is different from the value 1 (H1_1). This test was performed using the Student’s t-test with a type I error set at 0.05. A rejection of H0_1 may be interpreted as a selection of alleles in favor of *P. toxostoma* or *T. souffia*. The second hypothesis was related to the allelic association to form genotypes in panmixia. The hypothesis H0_2 indicates that the genotypes are in Hardy–Weinberg (HW) equilibrium, while H1_2 indicates HW disequilibrium. We used the exact Hardy–Weinberg test (Weir, 1996), using the HWExactMat function of the HardyWeinberg package (Graffelman, 2015). Disequilibrium may correspond to several situations: an excess of heterozygous, an excess of homozygous, or an excess of homozygous loci linked to a species (*P. toxostoma* or *T. souffia*). We used the D chi-square statistic from the HWChisqMat function from the HardyWeinberg package (Graffelman, 2015) to identify the different situations. Statistical analyses were performed using R software version 3.6.3 (R Core Team, 2020).

### 2.8 Gene ontology (GO term) analysis on orthologous ENSDARGs

To perform overrepresentation of biological functions, we used the PANTHER classification system (http://www.pantherdb.org/) with a reference constituted by the 617 SNPs corresponding to 542 orthologous ENSDARGs. We further performed Fisher’s exact test and false discovery rate correction. Because significant differences could be found between individuals belonging to the first cohort >2 cm and those belonging to the cohort = 2 cm, two separate analyses were performed.

### 2.9 Ethics statement

All sampling and experimental protocols involving the fish in this study (*Telestes souffia* and *Parachondrostoma toxostoma*) were reviewed and approved by local regulatory agencies: the AFB (Agence Française pour la Biodiversité) and the DDT (Direction Départementale des Territoires) from Alpes-de-Haute-Provence, Hautes-Alpes and Vaucluse (authorization numbers 2007–573 and 2008–636), following national regulations.

## 3 Results

### 3.1 Selection of discriminant loci using the *P. toxostoma* orthologous ENSDARGs

**FIGURE 2:**
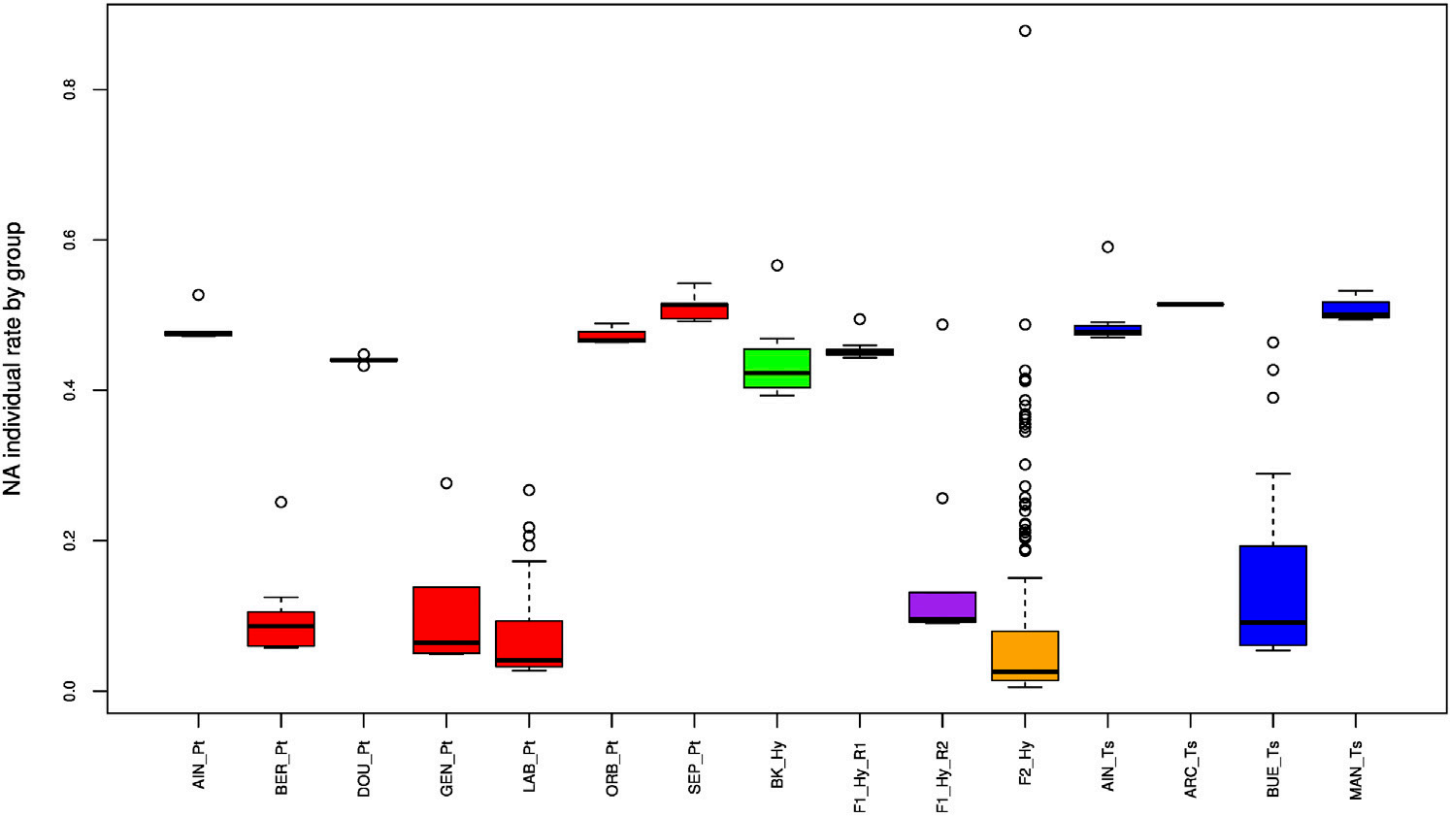
Boxplot of the missing data rate for the different groups (see Table 1). In red, *P. toxostoma* populations (AIN_Pt for Ain River, DOU_Pt for Doubs River, ORB_Pt for Orbieu River, BER_Pt for Berre River, GEN_Pt for genitors, LAB_Pt for laboratory specimens, SEP_Pt for Serre-Ponçon Lake); in blue, *T. souffia* populations (AIN_Ts for Ain River, ARC_Ts for Archidiacre site on Durance River, BUE_Ts for Buech River, MAN_Ts for Manosque on Durance River); in purple, F1_Hy_R1 for first run of F1 laboratory specimens, and F1_Hy_R2 for second run of F1 laboratory specimens; in orange, F2_Hy for F2 specimens; in green, BK_Hy for backcrossed laboratory specimens between F1 and *P. toxostoma* laboratory specimens.

Based on the parameters described in the Material and Methods, we conserved all the individuals reported in Table 1. The initial data set included 369 individuals related to 15 groups and 5,289 SNPs. The repeatability of 34 specimen replicates (DNA and tissue) within a run and between runs was verified, and 18 replicates were removed (see Section 2). Figure 2 displays the missing (NA) individual rate by a group. Two clusters could be observed: the first one was constituted by samples yielding around 50% NA and the other one by samples yielding 10% NA. This was clearly related to the sequencing depth of the two runs. We removed one specimen that presented a very high level (>80%) of NA. This individual was observed in the deeper sequencing run (second run).

**FIGURE 3:**
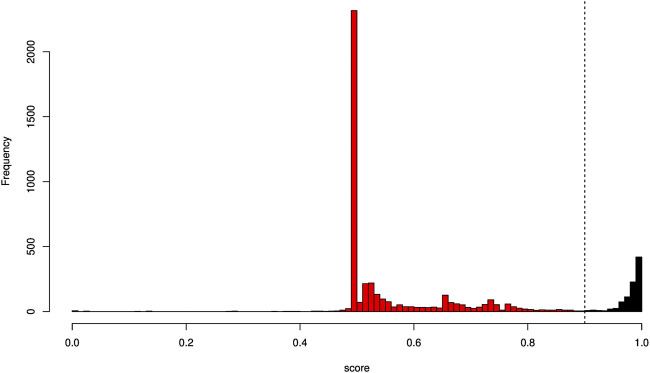
Histogram of SNP discriminating score. The *x*-axis represents the score values; the *y*-axis represents the absolute frequency of SNP. The vertical dotted line indicates the threshold (0.9) considered to define an SNP as discriminant. SNPs whose scores were higher than 0.9 were conserved and qualified as discriminant (in black). SNPs with scores less than or equal to 0.9 (in red) were discarded.

We then calculated the discriminant score for each SNP considering the two species *P. toxostoma* and *T. souffia*. The SNP discriminant score ranged from 0 to 1. An SNP exhibiting a score of 1 corresponds to a diagnostic locus. When the score is less than 1 and higher than 0.5, the loci is considered semi-diagnostic, and a score less than or equal to 0.5 refers to a non-diagnostic locus. We used a stringent cut-off of 0.9 (Figure 3) and restricted the dataset to the 908 SNPs considered discriminant loci. The discriminant SNPs represented 17% of the 5,289 SNPs. These SNPs were slightly related to chromosomes (Chi^2^ = 42.14, df = 24, *p* = 0.011), with a higher rate of discriminant SNPs for chromosomes Chr5 and Chr20 (see Supplementary Figure S1 for more information). However, chromosome Chr3 had a lower rate of discriminant SNPs.

**FIGURE 4:**
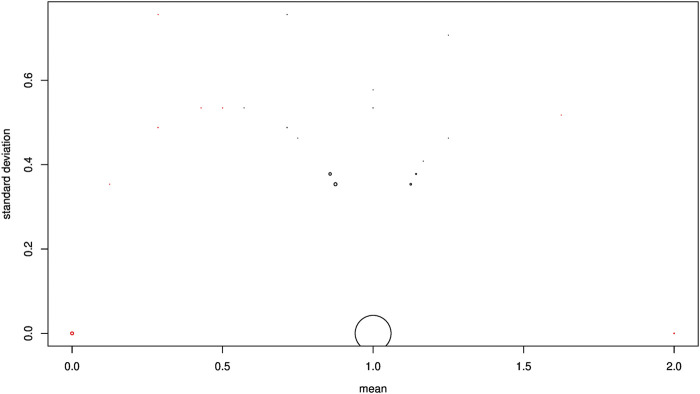
Volcano eruption plot. Each SNP is represented by a dot. The abscissa shows the mean genomic score calculated on F1 specimens; the ordinate shows the standard deviation of genomic scores calculated on F1 specimens. A coordinate point (x = 1, y = 0) is in perfect agreement with the Hardy–Weinberg equilibrium, indicating that an F1 individual is a heterozygote. Any point that deviates from (x = 1, y = 0) deviates from the HW equilibrium, indicating at least one homozygous F1 individual. All SNPs that deviate significantly from 1 on the *x*-axis are indicated in red; these SNPs were then discarded from the rest of the study. When several SNPs had the same coordinate, the size of the point was proportional to the number of SNPs.

**FIGURE 5:**
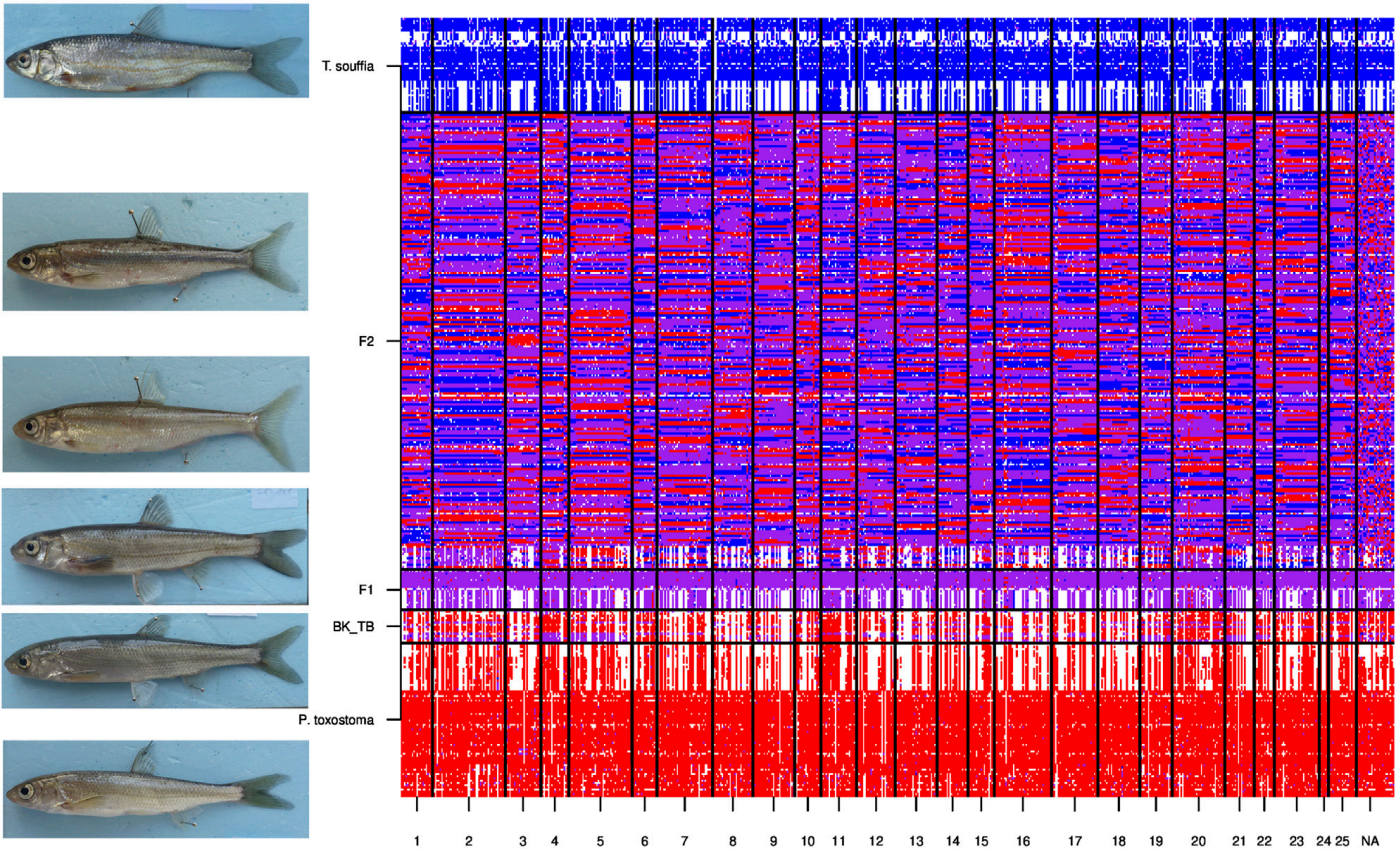
Genetic architecture of nuclear DNA SNPs for hybrid and parental populations. Lines represent specimens belonging to the six groups: respectively from bottom to top: *P. toxostoma*, Backcrosses (*P. Toxostoma* X F1-hybrids), F1-hybrids, F2-hybrids and *T. souffia*. Columns correspond to the 641 discriminants SNPs following chromosoms numbers (1 to 25, NA for not attributed). Each intersection line X column corresponds to a genotype. This genotype is indicated in red for homozygous *P. toxostoma*, in blue for homozygous *T. souffia*, in purple for heterozygous, and in white for missing genotype.

To validate the Mendelian inheritance of discriminant SNPs, we analyzed each 908 selected loci in F1-hybrids. As 204 SNPs were missing for the whole F1-hybrids, these were removed. The volcanic eruption plot (Figure 4) shows, for each SNP, the mean score value for the F1-hybrids, and the corresponding standard deviation on the ordinates. A majority of loci (507/704) presented the expected F1 values (i.e., heterozygous genotypes for all loci), with a mean score of 1 on the *x*-axis and a standard deviation of 0 on the *y*-axis. The mean score for 63 of the 704 loci deviated significantly from 1 (*t*-test, *p* < 0.05), and was removed. Finally, we obtained an intermediate data set constituted of 641 SNPs and 350 individuals. The rest of the SNP corresponded to shared alleles between the two species. The 641 SNPs corresponded to 560 *P. toxostoma* orthologous ENSDARGs, showing a decreasing exponential distribution. Indeed, 492 of the ENSDARGS were related to one SNP, 58 to two SNPs, 7 to three SNPs, and 3 to four SNPs. The genetic architecture map for pure individuals of each species, F1-hybrids, F2-hybrids, and backcross is presented in Figure 5.

### 3.2 Quantification of genomic dilution for the different hybrid specimens

**FIGURE 6:**
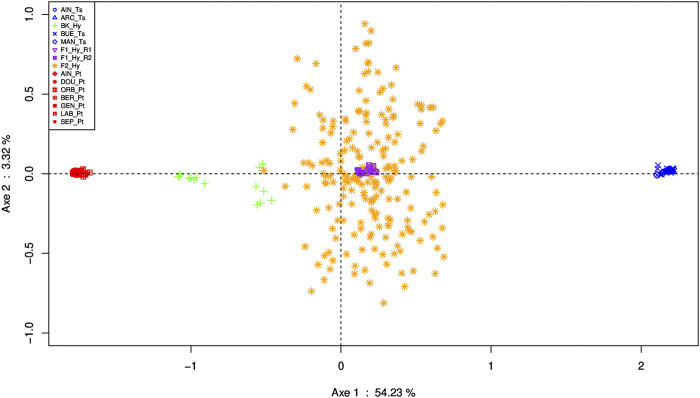
Specimen representation on the main PCA plane performed on SNP discriminant data, with standardization as proposed by (Patterson et al., 2006). In red, *P. toxostoma* populations (AIN_Pt for Ain River, DOU_Pt for Doubs River, ORB_Pt for Orbieu River, BER_Pt for Berre River, GEN_Pt for genitors, LAB_Pt for laboratory specimens, SEP_Pt for Serre-Ponçon Lake); in blue, *T. souffia* populations (AIN_Ts for the Ain River, ARC_Ts for the Archidiacre site in Durance River, BUE_Ts for the Buech River, MAN_Ts for Manosque on the Durance River); in purple, F1_Hy_R1 for the first series of F1 laboratory samples, and F1_Hy_R2 for the second series of F1 laboratory samples; in orange, F2_Hy for F2 samples; in green, BK_Hy for laboratory samples backcrossed between F1 and *P. toxostoma*.

PCA based on 350 individuals and 641 SNPs clearly displayed phylogenetic information on the first axis (54.24%, *p* < 10^–6^) (Figure 6). Indeed, the specimens belonging to the pure species are defined by the first axis: groups belonging to *P. toxostoma* species DOU_Pt, SEP_Pt, AIN_Pt (corresponding to parapatric populations), BER_Pt, ORB_Pt (corresponding to allopatric populations), GEN_Pt (from the Durance River), and LAB_Pt (corresponding to *P. toxostoma* laboratory stock); and those belonging to *T. souffia* ARC_Ts, MAN_Ts, BUE_Ts, and AIN_Ts. The F1-hybrids displayed a central position with low dispersion, indicating a classical 50/50 genomic composition. Similarly, the F2-hybrids displayed a central position but showed a wide range between the two species, indicating genomic heterogeneity due to parental dilution. On the other hand, as expected, the backcross specimens occupied an intermediate position between the F1-hybrid and *P. toxostoma* specimens.

These results were in agreement with the STRUCTURE analysis with k = 2 (Supplementary Figure S2). The groups belonging to the *P. toxostoma* species were assigned to one cluster, and those belonging to *T. souffia* were assigned to another cluster. The Q-score of the F1-hybrid specimens for the *P. toxostoma* and *T. souffia* clusters presented a mean of 0.507 and 0.493, respectively, and a standard error of 0.008, indicating a similar genotype. The F2-hybrid displayed a mean of 0.513 (0.487) and a standard error of 0.06, indicating a mosaic of genotypes. Finally, the backcrossed specimens yielded a mean of 0.866 (0.134) and a standard error of 0.122, indicating a genomic introgression to *P. toxostoma*.

### 3.3 Establishment of endogenous selection in F2 specimens

**FIGURE 7:**
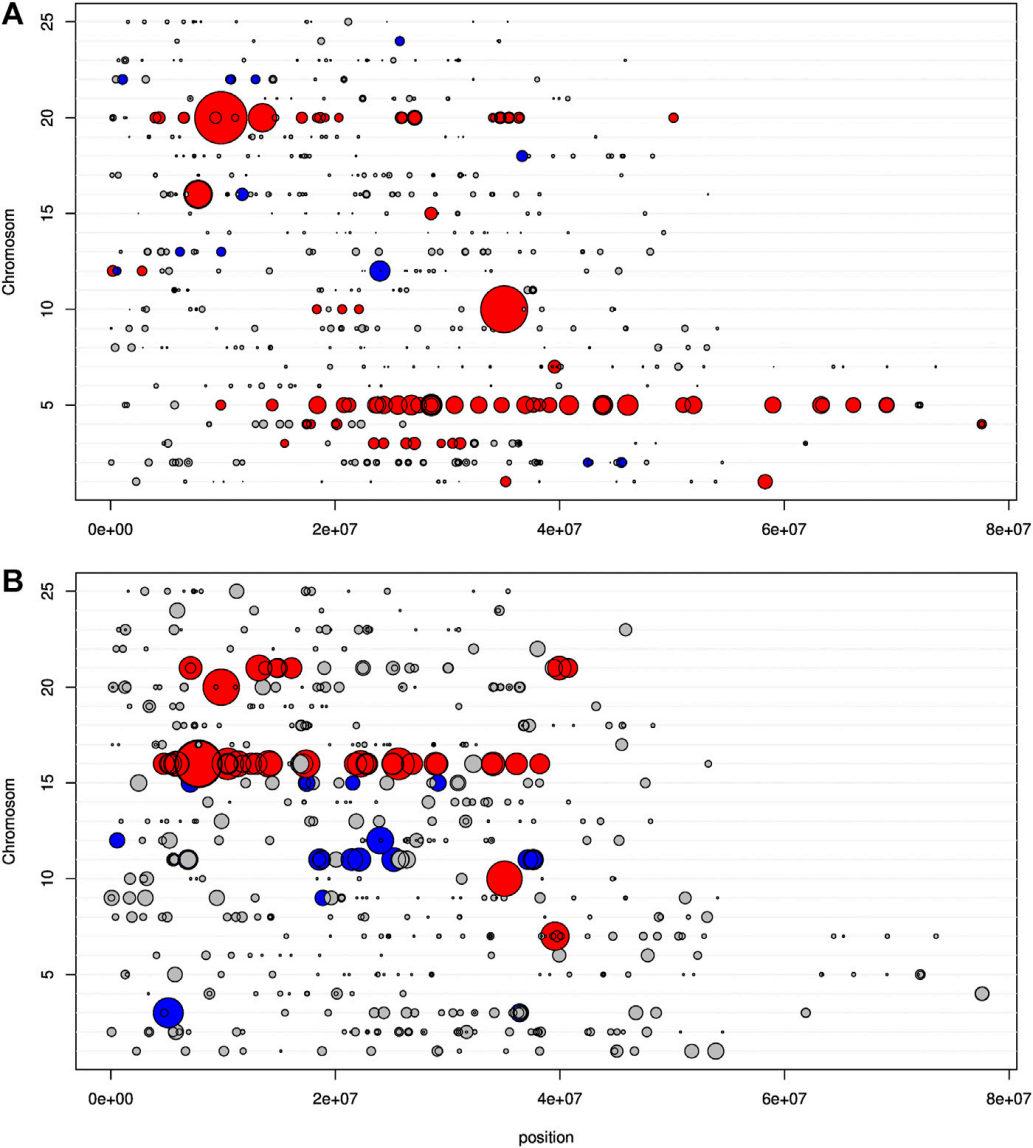
Representation on *D. rerio* chromosomes of the t-statistic expressing the deviation from 1 for the mean genotypic score, calculated on F2 individuals. In grey, non-significant deviation; in blue, significant deviation toward *T. souffia*; in red, significant deviation toward *P. toxostoma*. The size of the dot is proportional to the deviation. **(A)** Specimens with a size >2 cm. **(B)** Specimens with a size of 2 cm.

We next considered 204 F2-hybrids specimens and the 617 SNPs among 641 associated with *Danio rerio* chromosomes. However, to explore a potential postzygotic barrier (lineage sorting of particular genomic combination), we first analyzed the cohort of 166 F2 individuals who survived (>2 cm) and, second, the cohort of 38 F2 individuals who died very young (=2 cm). Of the 617 discriminant SNPs, only 86 SNPs (13.94%) were significantly (*p* < 0.05) different from allele frequencies *p* = *q* = 0.5, indicating an increase of allele frequencies toward one species (Figure 7A). Interestingly, some genomic regions were clearly weighted toward *P. toxostoma,* especially in the chromosomes Chr5 and Chr20, while those weighted toward *T. souffia* were more sparse. The allele frequency ranged from 0.55 to 0.84 for *P. toxostoma* and 0.55 to 0.65 for *T. souffia*.

In the second test, we tested the HW equilibrium for each locus. We found that a majority of loci (563/617, 91.25%) were in HW equilibrium. The loci departing from HW equilibrium mainly presented an excess of heterozygotes (47/617, 7.62%) (Supplementary Figure S3A).

**FIGURE 8:**
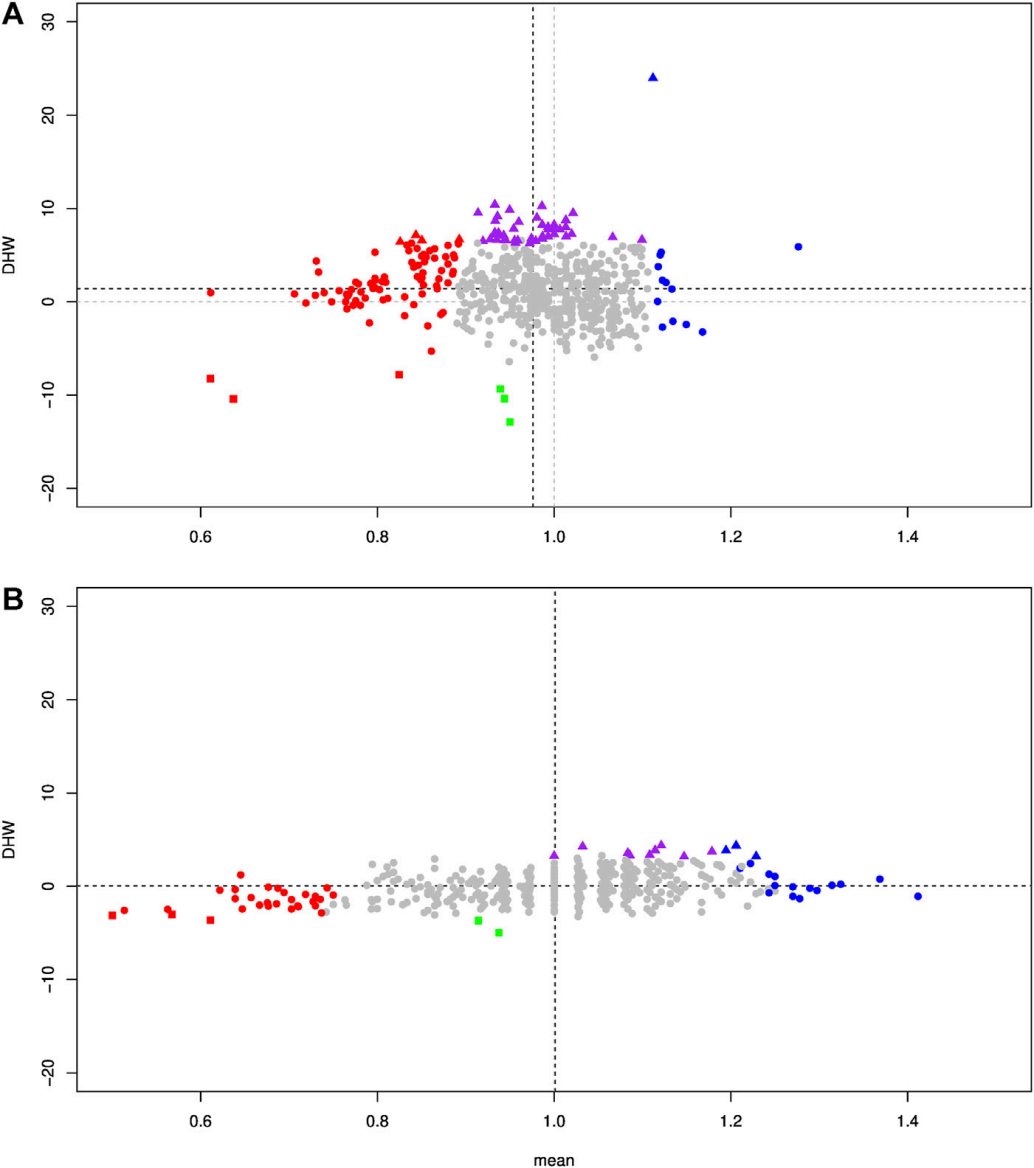
Biplot of the DHW statistic of the Hardy–Weinberg deviation as a function of the t-statistic expressing the deviation from 1 for the allelic composition, calculated on F2 individuals. Grey circles represent the SNPs that were not significant for both tests. Red circles and blue circles represent SNPs in HW equilibrium, for which allele composition deviates significantly from 1 toward *P. toxostoma* and *T. souffia*, respectively. Red and blue triangles represent SNPs in Hardy–Weinberg disequilibrium (excess heterozygote) for which allelic composition deviates significantly from 1 toward *P. toxostoma* and *T. souffia*, respectively. Purple triangles represent SNPs in Hardy–Weinberg disequilibrium (excess heterozygote) whose allele composition does not deviate significantly from 1. Red squares represent SNPs in Hardy–Weinberg disequilibrium (excess *P. toxostoma* homozygote) whose allelic composition deviates significantly from 1 toward *P. toxostoma*. Green squares represent SNPs in Hardy–Weinberg disequilibrium (excess homozygote for both species) for which allelic composition does not deviate significantly from 1. **(A)** Specimens with a size >2 cm. **(B)** Specimens with a size of 2 cm.

Figure 8A shows the relationship between these two phenomena, that is, the deviation of the allele frequencies and HW disequilibrium. A large majority (475/617 loci, 76.99%) displayed a neutral evolution, with HW equilibrium and no deviation in allele frequency, indicating high genomic compatibility between the two genomes. We could consider the absence of deviation proportion as an estimate of the porosity between the two species. Furthermore, 45 loci (7.29%) presented no deviation in allele frequency between the two parental species but were in HW disequilibrium. 42 loci (6.81%) presented an excess of heterozygous genotypes (purple triangle, Figure 8A), indicating a heterozygous advantage. These loci were mainly related to three chromosomes: Chr3, Chr9, and Chr15 (Supplementary Figure S3A). In contrast, only three loci (0.49%) presented a heterozygous disadvantage (green square, Figure 8A). Among the 84 loci (13.61%) presenting a higher frequency toward *P. toxostoma* (red color in Figure 8A), 76 were in HW equilibrium (red circle in Figure 8A), 4 presented excess homozygous *P. toxostoma* genotypes (red square in Figure 8A), and 4 presented an excess of heterozygous genotypes (red triangle in Figure 8A). Among the 13 loci (2.11%) presenting a higher frequency toward *T. souffia* (blue color in Figure 8A), 12 displayed HW equilibrium (blue circle in Figure 8A), and 1 presented an excess of heterozygous genotypes (blue triangle in Figure 8A).

Next, we focused our analysis on the cohort of F2s who died very young (=2 cm). This analysis displayed a very different pattern, as shown in Figure 7B. Of the 617 discriminant SNP, only 59 SNP (9.56%) were significantly different from allele frequencies *p* = *q* = 0.5. We detected genomic regions weighted toward *P. toxostoma* (chromosomes Chr16 and Chr21) and spotted regions (Chromosomes 7, 10, and 20), combined with genomic regions weighted toward *T. souffia* (Chromosome 11) and a spotted chromosome (Chr15). This result clearly indicated a selection toward *T. souffia* alleles in Chromosomes 11 and 15. However, the HW disequilibrium was less pronounced for this cohort than in the cohort >2 cm (Figure 8B, Supplementary Figure S3B), indicating that most loci were in HW equilibrium. Indeed, 9 loci (purple triangle) presented an excess of heterozygous genotypes, and 2 (green square) presented a heterozygous disadvantage.

The porosity between the two genomes appears to be high but not complete. Indeed, we observed the signature of endogenous selection in favor of both species, mostly toward *P. toxostoma*. This pattern was particularly marked on the cohort > 2 cm indicating the presence of filters limiting the genome porosity during fish development. Considering F2 genotypes, we also observed a positive selection in favor of heterozygotes corresponding to an increase in the genomic porosity by maintaining high genomic compatibility.

### 3.4 Gene function associated with the two F2 cohorts

Of the 142 genes that presented an atypical pattern, that is, deviation of allele frequencies and/or HW disequilibrium, in the cohort >2 cm, two categories were well represented. The 76 genes that presented allelic frequency toward *P. toxostoma* and HW equilibrium (red circle in Figure 8) were implicated in positive regulation of developmental processes (GO:0,051,094; 5/7; *p* = 5.08E-03), regulation of organelle organization (GO:0,033,043; 6/17; *p* = 2.67E-02, sptbn2 and cntrl), especially in the cytoskeleton (GO:0,051,493; 5/14; *p* = 4.14E-02), chromosome organization (GO:0,051,276; 5/14; *p* = 4.14E-02, mcm3l and recql), cell cycle (GO:0,007,049; 5/14; *p* = 4.14E-02, ptpa), and negative regulation of transcription, DNA-templated (GO:0,045,892; 5/15; *p* = 5.08E-02, hif1ab). The 42 genes that presented an excess of heterozygous genotypes (purple triangle in Figure 8) were implicated in several developmental functions, such as innate immune response (GO:0,045,087; 2/4; *p* = 7.07E-02, stat1b, ifih1), pronephros development (GO:0,048,793; 2/4; 7.05E-02, prkcsh), and liver development (GO:0,001,889; 2/5; 9.40E-02, leg1a).

Of the 70 genes that presented an atypical pattern in the cohort = 2 cm, two categories were well represented. Indeed, 35 corresponded to allelic frequency toward *P. toxostoma* and HW equilibrium (red circle in Figure 8), and 15 corresponded to allelic frequency toward *T. souffia* and HW equilibrium (blue circle in Figure 8). The first cluster was implicated in endosome to lysosome transport (GO:0,008,333; 2/2; *p* = 3.76E-03, trak1a), mitochondrion organization (GO:0,007,005; 2/2; *p* = 3.76E-03, ulk1b), vesicle cytoskeletal trafficking (GO:0,099,518; 2/5; *p* = 1.34E-02, myo1eb), and regulation of protein depolymerization (GO:1,901,879; 2/5; *p* = 1.71E-02, nestin). The second cluster was implicated in positive regulation of potassium ion transport (GO:0,043,268; *p* = 1.40E-02, wnk3) and positive regulation of sodium ion transport (GO:0,010,765; *p* = 1.40E-02, wnk2).

## 4 Discussion

### 4.1 The importance of studying new hybrid models in laboratory: reproductive isolation versus genetic incompatibility

The importance of performing crosses in the laboratory to study speciation/hybridization is no longer in question (Liu et al., 2016; White et al., 2020). However, some biological models, such as many European river cyprinids, require several years of rearing before breeding, especially F2-hybrid specimens, and typically survival rate data is based on F1-hybrid specimens (Philippart and Berrebi, 1990; Nzau Matondo et al., 2007). At first sight, we expected a very strong genetic incompatibility between these two species because the reproductive isolation seemed particularly important given the anecdotal number of hybrids (*n* = 2) observed in the field for more than 25 years (Dubut et al., 2010; Dubut et al., 2012). However, we have observed a strong and repeatable genomic compatibility in our farms. It is, therefore, crucial to describe and quantify this compatibility in order to explain the decoupling between the reproductive isolation observed in the wild and the strong genetic compatibility in the laboratory of two such divergent species living in sympatry.

### 4.2 F1 individuals are the cornerstone in the calibration of discriminant single-nucleotide polymorphisms

The first strength of our work was that we were able to select discriminant SNPs from different parental populations. This allowed us to take into account the polymorphism of each species by having an *a priori* knowledge of the existence of hybrid individuals, as they have been the subject of numerous studies (Dubut et al., 2010; Dubut et al., 2012; Corse et al., 2015). The second strength of our work was the use of F1 individuals, which allowed us to select the discriminant SNPs of the parental populations that were indeed in the heterozygous state in the F1s. We found that a large majority of these SNPs (507/704, 72.02%) were consistently heterozygous in F1s. Among them, each of the 415 SNP was associated with a single transcript (conveniently named orthologous ENSDARG), and their distribution across the *Danio* transcriptome/genome was not completely random (Chi^2^ = 42.14, df = 24, *p* = 0.011). Two explanations can be formulated: The first is that our *P. toxostoma* transcriptome has non-random areas for which we did not obtain the genes, which results in missing some SNPs. The second is that by using the position of the *D. rerio* genes, we assumed that the position of the genes on the chromosomes studied had not changed. It is clear that large regions of synteny exist in Cyprinidae (Liu et al., 2017a; Chen et al., 2019; Lei et al., 2021); however, there are also shuffles (Liu et al., 2017b; Chen et al., 2019). If these shuffles are not random, then they may explain a preferential association between SNPs, ENSDARGs, and chromosomes.

### 4.3 Remodeling a hybrid genome: Allelic frequency and Hardy–Weinberg disequilibrium in F2s

The selected SNPs show a very strong discriminating power. Whether using PCA or assignment by STRUCTURE, we clearly separated the different genetic groups: *P. toxostoma*, *T. souffia*, F1-hybrids, F2-hybrids, and backcross. We were thus able to focus on the F2-hybrid individuals that survived beyond 2 cm (i.e., >2 cm). The study of the 617 SNPs showed that a large majority (475/617 loci, 76.99%) were evolving under a panmictic model; that is, the alleles of these genes were interchangeable. The set of F2 individuals that we analyzed constitutes a sorting of compatibility between our two species after a succession of pre- and post-zygotic stages, such as gamete recognition, fertilization, embryonic development, and larval metamorphosis. Two phenomena are underlined by our results.

The first phenomenon is related to the significant deviation of the allelic frequency (*p*≠q, directional selection) in the F2 specimens, notably in favor of *P. toxostoma*, although it was also present in favor of *T. souffia*. The fact that the extreme majority of these loci were in HW equilibrium implies an unbalanced production of gametes, whose association would be random in aquatic environments. Moreover, we note that certain genomic regions, such as chromosomes Chr5 and Chr20, seemed to display this phenomenon. If these regions are indeed regions of synteny between *D. rerio* and the two other Cyprinidae species (*P. toxostoma* and *T. souffia*), then the structural incompatibility may involve a problem of chromatin interaction in these regions. This phenomenon could be linked with the asymmetric expression of alleles in cyprinid hybrid lineages (Ren et al., 2016; Ren et al., 2019). Furthermore, in 2-cm-sized individuals with high mortality, we were able to observe particular genomic signatures displaying this phenomenon but involving different genomic regions (chromosomes Chr16 and Chr21).

The second is related to HW disequilibrium, essentially genes with frequencies *p* = *q* = 0.5. In this case, we believe that this phenomenon is the result of successive sorting during the different stages of development, which will continue until adulthood. Some genomic combinations will be favored (heterozygous advantage and stabilizing selection) while others will be selected against (disruptive selection).

### 4.4 What are the functions of these genes?

We were able to identify two broad categories of genes. Genes that tended to be more *P. toxostoma*-like, while being in HW equilibrium, were involved in developmental regulatory processes and cytoskeletal organization but were also involved in chromosome organization and DNA regulation, which suggests not only that epigenetic factors are of great importance in the development and maintenance of the hybrids but also that this stability is more related to *P. toxostoma* alleles. Genes that showed a heterozygous advantage were involved in immune response and kidney/liver development.

Individuals with a 2-cm size, which died almost at the same time, had common characteristics, notably in endosome to lysosome transport and cytoskeletal trafficking, with allele frequency toward *P. toxostoma*. However, these F2-hybrids presented differences in genes involved in mitochondrion organization and positive regulation of potassium and sodium. In conclusion, the ‘genomic landscape’ of F2-hybrids is a mixture of genes whose alleles are fully compatible, at least under laboratory conditions, and genes whose alleles show different evolutionary trajectories (Martin and Jiggins, 2017).

### 4.5 Potential shortcomings and limitations

Our study showed that two endemic species belonging to two different genera had very high genomic porosity with some disadvantaged genomic combinations. We were able to identify certain genes and biological functions associated with these disadvantages. Currently, it is not possible to know whether these patterns result from a structural problem related to the DNA sequences due to allele differences (*T. souffia* versus *P. toxostoma*) or the regulation of these genes. In the first case, it would be interesting to use the complete set of SNP markers to describe the different selections between gene and intergenic regions. However, the use of the *Danio rerio* genome remains limited because of the low similarity between *Telestes/Parachondrostoma* sequences and the *Danio rerio* genome, and a reference *Telestes/Parachondrostoma* genome would be necessary. In the second case, it would be necessary to work on RNA sequencing to analyze differential gene expression (DGE) in order to estimate the effects of dominance, co-dominance, and recessivity of the different alleles belonging to *T. souffia* and *P. toxostoma*.

Moreover, it will be interesting to test the parameters involved in the overlapping breeding because the reciprocal crosses (i.e., one female *P. toxostoma* and one male *T. souffia*) did not produce hybrid individuals. Currently, we don’t know if this result is due to a genetic incompatibility or a handling problem because nine backcrosses were obtained by crossing female *P. toxostoma* and male F1-hybrids, with *P. toxostoma* mitochondrial DNA (i.e., *T. souffia* for F2-hybrids). These results may be the consequence of imprinting effects.

### 4.6 Can we expect the emergence of a *P. toxostoma*–*T. souffia* hybrid zone?

The results obtained in the laboratory show that the main barrier is not related to genomic incompatibility. We, therefore, deduce that the barriers which currently strongly limit the emergence of a *P. toxostoma*–*T. souffia* hybrid zone are exogenous. To establish the emergence of a *P. toxostoma*–*T. souffia* hybrid zone, the pre-zygotic barrier would have to be broken between the two species (Crispo et al., 2011; Grabenstein and Taylor, 2018), forcing them to reproduce on the same spawning grounds at the same time, as observed for other cyprinids (Balon, 1992). *P. toxostoma* specimens reproduce essentially during the month of April until the beginning of May in the range of water temperature from 11 to 13°C in the Buech tributary of the Durance, France (Costedoat et al., 2007), whereas *T. souffia* specimens reproduce in June, in water temperatures from 10 to 14°C. Thus, an increase in river temperature in late spring, coupled with a decrease in river flow, could be the trigger for natural hybridization between these two species, as in a faster warming environment, the breeding periods would overlap. It can then be assumed that the embryonic development of the hybrids would exhibit *T. souffia*-like embryonic development. Indeed, the embryonic development is 7 days at 13.9°C for *T. souffia*, while the embryonic development of *P. toxostoma* is 8 days at 16°C and 10 days at 14°C (Gozlan et al., 1999). However, in adulthood, the advantage would be in favor of *P. toxostoma*, because the optimal adult temperature range for *P. toxostoma* is 16–25°C, while it is between 10 and 18°C for *T. souffia* (Tissot and Souchon, 2010). There would thus be adaptive trade-offs related to growth, reproduction, feeding, and river temperature (Daufresne et al., 2004), which could be implemented by the selection of new hybrid combinations (exogenous selection). Obviously, these trends would be evolutionary compromises depending on the genes presenting genetically compatible alleles (endogenous selection) and the density of each species. It would not be surprising to observe an asymmetrical introgression in such cyprinid fish hybrid zones (Crespin et al., 1999). With climate change, future studies should focus on the establishment of new hybrid zones involving a genomic mixing of several endemic and/or introduced species (common garden or *in natura*). It will be particularly interesting to decipher the gene expression of F1 and F2 hybrid specimens considering their genomic combination, especially for the F2 specimens (evolutionary novelties, transgressive segregation, etc.). Common garden experiments will be associated with field sampling in order to detect new hybrid specimens that underwent the filter of environmental selection. They will have to better understand the part related to endogenous selection from those related to exogenous selection. These topics are essential in the context of global warming and accelerated environmental changes.

## Data Availability

The original contributions presented in the study are publicly available. This data can be found here: https://www.ncbi.nlm.nih.gov/sra?linkname=bioproject_sra_all&from_uid=863842, accession number PRJNA863842.
