# Supplementary material for: Breaking the reproductive barrier of divergent species to explore the genomic landscape
Source: Front Genet. 2022 Sep 23;13:963341. doi: 10.3389/fgene.2022.963341 (PMC9538152; doi:10.3389/fgene.2022.963341)
Supplement: Supplementary file 1 [file Table1.DOCX]

>Telestes_souffia

ATGGCAAGCCTACGAAAAACCCACCCACTAATAAAAAT

CGCTAATGACGCACTAGTCGACCTCCCAACACCATCTAATATTTCAGTAATATGAAACTT

CGGTTCTCTTCTAGGATTATGTTTAATTACCCAAATCCTAACAGGATTATTCTTAGCCAT

GCACTATACCTCTGACATCTCAACCGCATTCTCATCCGTAACCCACATCTGCCGAGACGT

CAACTACGGCTGACTTATCCGGAGCCTACATGCTAACGGGGCATCCTTCTTCTTCATCTG

CCTTTACATGCACATCGCACGGGGCCTGTATTATGGGTCATACCTTTATAAAGAAACCTG

AAGCATTGGTGTGGTTCTATTCCTTCTGGTTATGATGACAGCCTTCGTCGGCTACGTTCT

TCCATGAGGCCAAATATCCTTTTGAGGCGCTACCGTAATTACGAATCTCCTCTCAGCAGT

CCCATACATGGGAGATACCCTTGTTCAGTGAATCTGAGGCGGTTTCTCAGTAGACAATGC

AACTCTTACACGGTTCTTCGCATTCCACTTCCTCCTGCCATTTGTCATCGCCGGCGCAAC

CATTCTGCACCTATTATTCTTACACGAAACGGGATCAAACAACCCAGCCGGACTAAATTC

CGACGCGGACAAGATTTCTTTCCACCCATACTTCTCATATAAGGACCTTCTTGGCTTTGT

GGCAATATTGCTAGCCCTCACCTCTCTAACCTTATTCTCCCCGAACCTCTTAGGTGACCC

GGAGAACTTCACCCCAGCAAACCCACTCGTAACTCCTCCACATATCCAGCCGGAGTGGTA

CTTCTTGTTTGCCTACGCCATCCTCCGATCTATCCCAAATAAGCTAGGAGGGGTTCTTGC

ACTACTGTTCAGCATCCTGGTGCTAATAGTCGTGCCAATTTTACACACGTCCAAACAACG

AGGACTAACTTTCCGCCCAGTGACTCAATTCCTATTCTGAACCCTTGTTGCAGATATATT

TATTTTGACGTGAATCGGAGGCATACCCGTAGAACACCCATATATTATTATCGGCCAAAT

CGCATCCGTCCTATACTTTGCACTCTTCCTCGTCCTTGTCCCACTAGCGGGATGGGTGGA

GAATAAAGCATTGAAATGAGCC

>Parachondrostoma_toxostoma

ATGGCAAGCCTACGAAAAACCCACCCCCTAATAAAAAT

CGCTAACGGCGCACTAGTCGACCTCCCAACACCATCTAATATCTCAGCAATGTGAAACTT

CGGGTCTCTCCTGGGATTATGTTTGATTACCCAAATCCTAACAGGGTTATTCTTAGCCAT

GCATTACACCTCTGATATCTCGACCGCATTCTCATCAGTAACTCACATCTGCCGAGATGT

TAACTACGGCTGACTTATCCGGAGCCTACATGCCAATGGAGCATCCTTTTTCTTCATCTG

TCTTTATATACATATCGCACGGGGCCTGTATTATGGCTCATACCTTTATAAAGAGACCTG

AAACATTGGTGTAGTCCTATTCCTTCTGGTTATAATAACAGCCTTTGTCGGCTACGTCCT

TCCATGGGGACAAATGTCCTTTTGAGGCGCTACCGTAATTACAAACCTCCTATCTGCGGT

CCCCTATATAGGGGATACCCTCGTTCAATGAATCTGAGGTGGATTCTCAGTAGACAATGC

AACTCTCACACGATTCTTCGCGTTCCACTTCCTACTACCATTTGTCGTTGCCGGCGCAAC

CATCCTACATTTATTGTTTTTACACGAAACGGGGTCGAATAACCCGGCCGGACTAAATTC

AGACGCAGACAAGATTTCCTTCCACCCGTATTTCTCATATAAGGACCTTCTTGGCTTTGT

GGCCATGTTACTAGCCCTTACCTCCCTAACATTATTTTCCCCCAACCTACTAGGTGACCC

GGAAAACTTCACCCCAGCAAACCCTCTCGTGACACCCCCGCATATCCAGCCGGAATGATA

CTTCTTATTTGCCTACGCCATTCTTCGATCTATCCCAAATAAACTAGGAGGGGTTCTTGC

ACTACTATTCAGCATTCTAGTGCTAATAGTCGTGCCGATTTTACACACATCCAAACAACG

AGGACTAACTTTCCGGCCAGTGACCCAATTCCTATTCTGAACCCTCGTCGCAGATATATT

TATTTTGACATGAATCGGGGGTATACCCGTAGAACACCCATATATTGTCATTGGCCAAGT

CGCATCCGTCCTATACTTTGCACTATTCCTCATTCTTGTCCCGCTAGCAGGATGGGTGGA

AAATAAAG
