# Supplementary material for: Breaking the reproductive barrier of divergent species to explore the genomic landscape
Source: Front Genet. 2022 Sep 23;13:963341. doi: 10.3389/fgene.2022.963341 (PMC9538152; doi:10.3389/fgene.2022.963341)
Supplement: Supplementary file 2 [file Table2.DOCX]

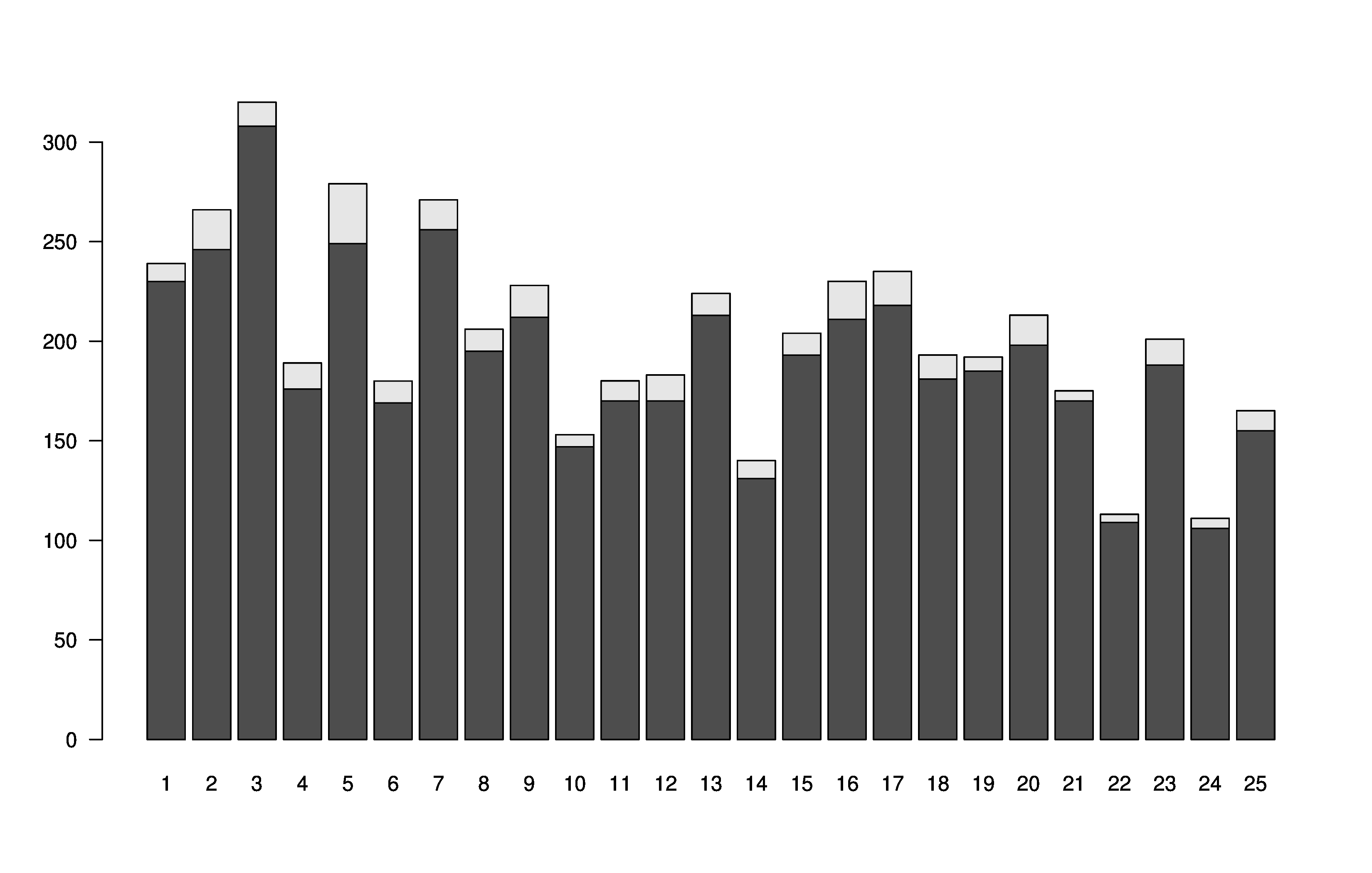
Supplementary Material Figure S1: Bar plot of the SNP absolute frequency for the 25 chromosomes. The black part refers to the non-discriminant SNPs and the grey part refers to the discriminant SNPs.
