# Supplementary material for: Breaking the reproductive barrier of divergent species to explore the genomic landscape
Source: Front Genet. 2022 Sep 23;13:963341. doi: 10.3389/fgene.2022.963341 (PMC9538152; doi:10.3389/fgene.2022.963341)
Supplement: Supplementary file 3 [file Table3.DOCX]

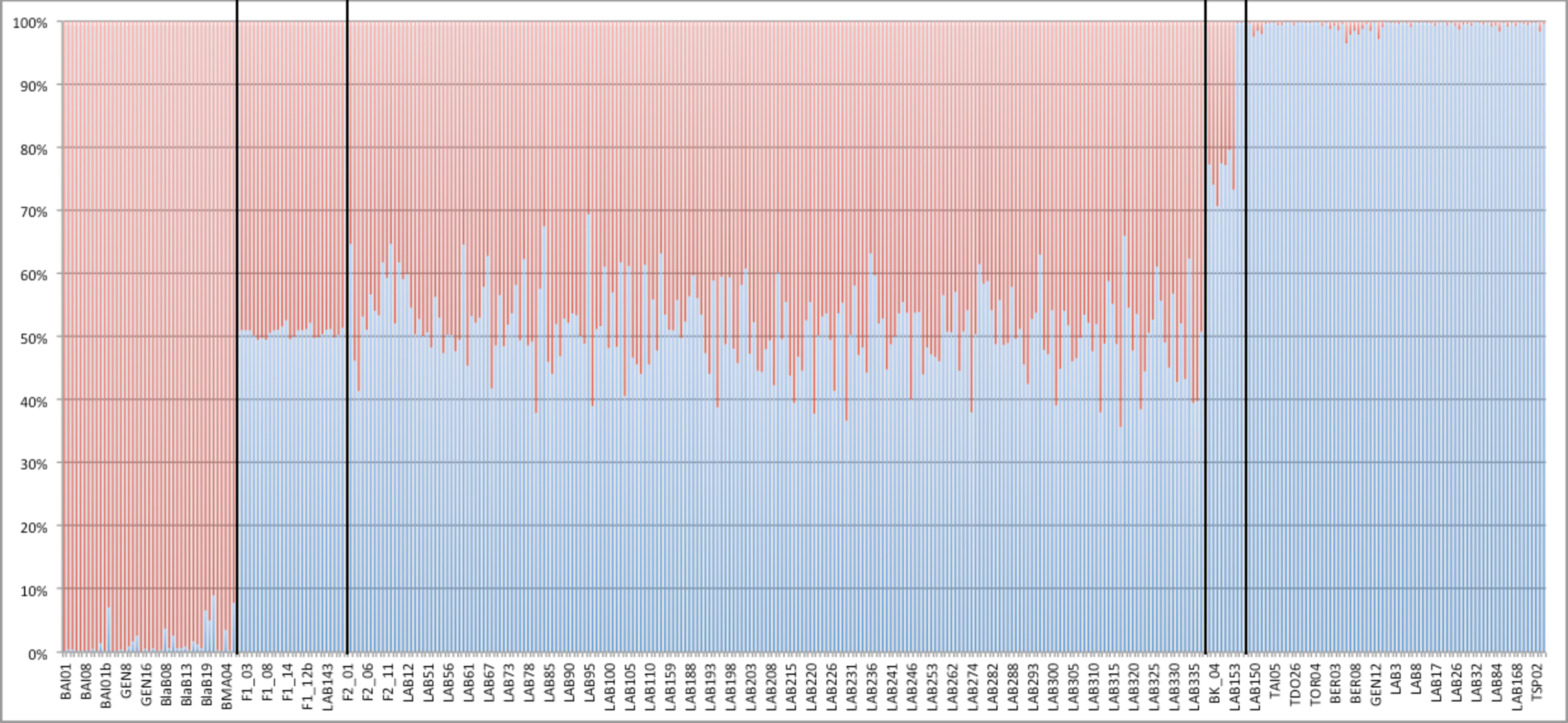


Supplementary Material Figure S2: Bayesian clustering analyses of SNPs using STRUCTURE for k=2 (*T. souffia* in red and *P. toxostoma* in blue). Each bar plot corresponds to a single individual, and the proportion of red colour and blue colour is equal to the probability to be assigned to *T. souffia* and *P. toxostoma*, respectively. Black lines separate the five different groups. From the left to the right: *T. souffia*, F1-hybrid, F2-hybrid, Backcrossed specimens, *P. toxostoma*.
