# Supplementary material for: Breaking the reproductive barrier of divergent species to explore the genomic landscape
Source: Front Genet. 2022 Sep 23;13:963341. doi: 10.3389/fgene.2022.963341 (PMC9538152; doi:10.3389/fgene.2022.963341)
Supplement: Supplementary file 4 [file Table4.DOCX]

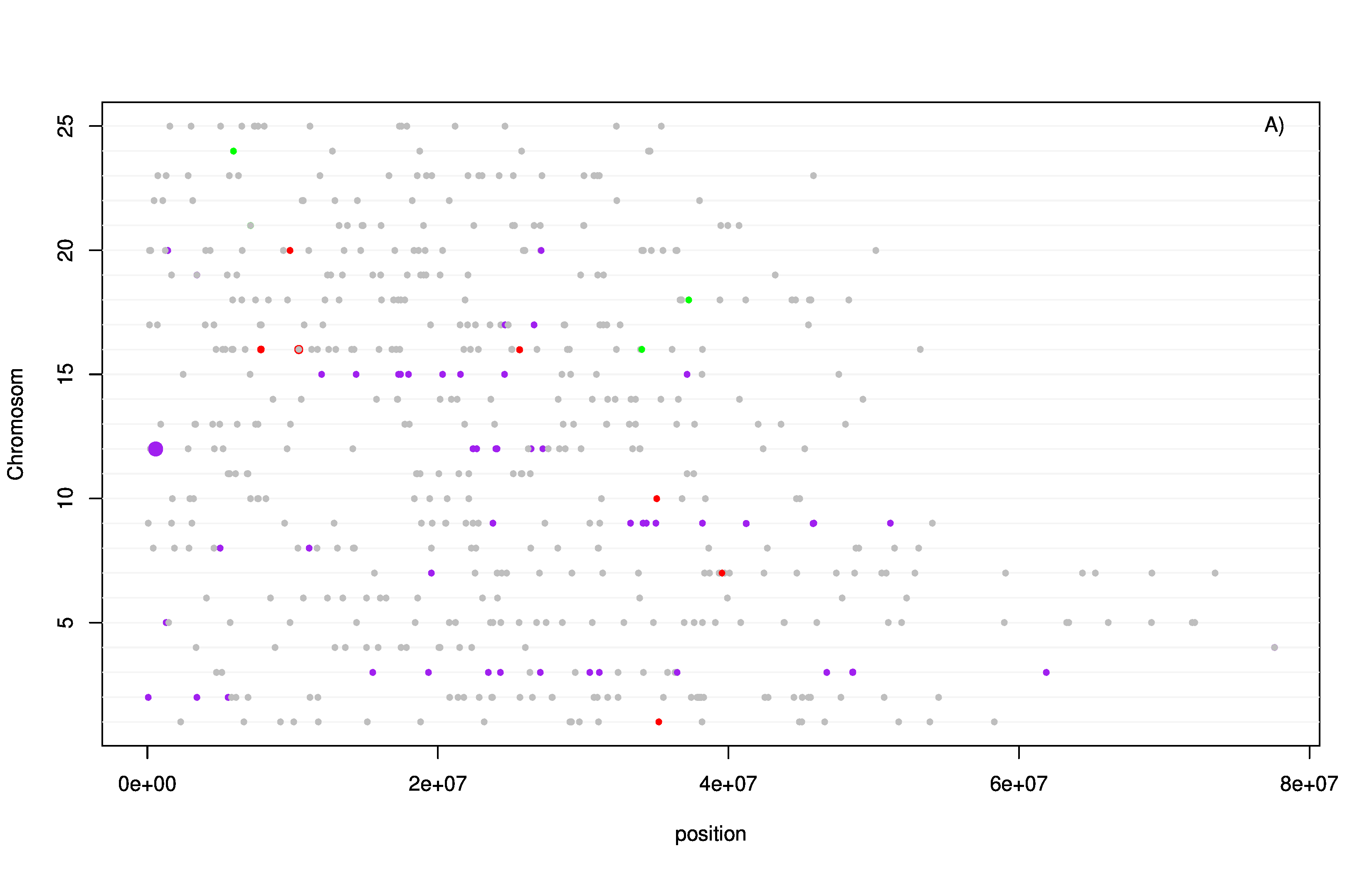

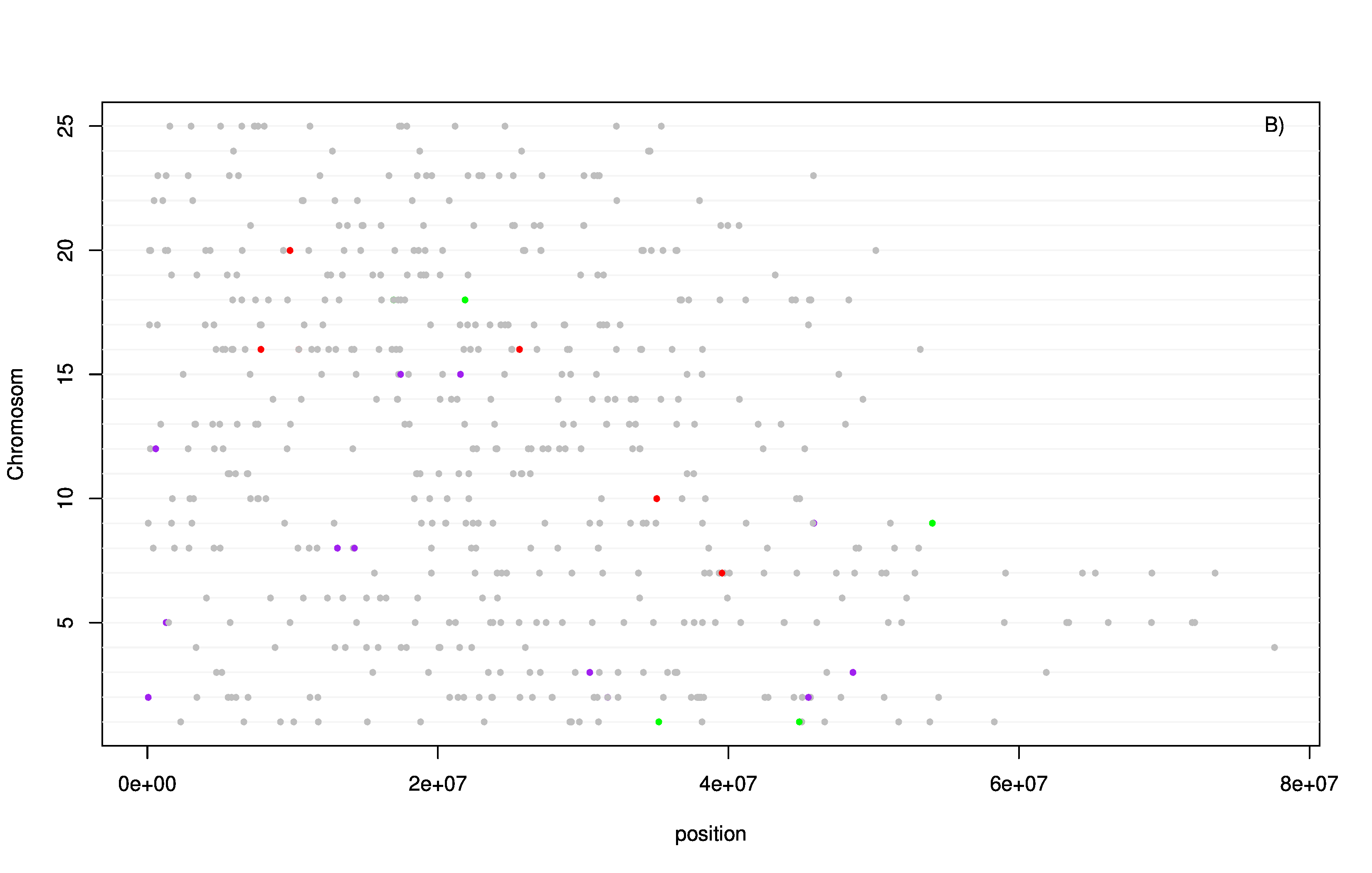


Supplementary Material Figure S3: Representation on chromosomes of the DHW (Deviation from Hardy-Weinberg Equilibrium) statistic expressing the deviation from the Hardy–Weinberg equilibrium for F2 individuals. In grey: non-significant deviation. In red: significant deviation toward *P. toxostoma* homozygotes. In green: significant deviation toward homozygotes for both species. In purple: significant deviation toward heterozygotes. The size of the dot is proportional to the deviation. (**A**) Specimens with a size >2 cm. (**B**) Specimens with a size of 2 cm.
